# Insights of Crosstalk between p53 Protein and the MKK3/MKK6/p38 MAPK Signaling Pathway in Cancer

**DOI:** 10.3390/cancers10050131

**Published:** 2018-05-03

**Authors:** Lorenzo Stramucci, Angelina Pranteda, Gianluca Bossi

**Affiliations:** Laboratory of Medical Physics and Expert Systems, Regina Elena National Cancer Institute, 00144 Rome, Italy; lorenzo.stramucci@gmail.com (L.S.); prantedaangelina@gmail.com (A.P.)

**Keywords:** p53, mutations, MKK3/MKK6, p38MAPK

## Abstract

TP53 is universally recognized as a pivotal protein in cell-cycle fate and apoptotic induction and, unsurprisingly, it is one of the most commonly hijacked control mechanisms in cancer. Recently, the kinase MKK3 emerged as a potential therapeutic target in different types of solid tumor being linked to mutant p53 gain-of-function. In this review, we summarize the delicate relationship among *p53* mutational status, MKK3/MKK6 and the downstream activated master kinase p38MAPK, dissecting a finely-tuned crosstalk, in a potentially cell-context dependent scenario that urges towards a deeper characterization of the different molecular players involved in this signaling cascade and their interactions.

## 1. Introduction

Mitogen activated protein kinases (MAPKs) are activated in response to a variety of stimuli, and mediate a plethora of cell processes to respond and adapt accordingly. In particular p38 mitogen activated protein kinase (p38MAPK), becomes activated in response to UV damage, oxidative stress, exposure to DNA damaging agents as well as growth factors and cytokines [1,2]. As for other MAPKs, p38MAPK activation orchestrates cellular response by modulating a wide variety of targets, such as protein kinases, phosphatases, cell-cycle regulators and transcription factors, including p53 [1,2,3,4].

TP53 is the epitome of tumor suppressors: because of its central role in responding to DNA damage it is referred to as guardian of the genome and is one of the most-commonly hi-jacked mechanisms in cancer. In fact, mutations in *p53* occur in about half of all solid tumors, resulting into inactivation of its physiological role or into gain of oncogenic activity. Additionally, the p53 exact role in tumor suppression has been recently re-calibrated to include effects other than acute DNA damage response [5,6,7,8].

Interestingly, the functional interaction between p53 and p38MAPK appears to be exerted at multiple levels. Indeed, being p53 a phosphorylatable target of this kinase, the *p53* status could directly contribute in the final outcome of p38MAPK signaling by a negative feedback loops in wild-type p53 cell-contexts, skewing the biological outcome of p38MAPK activation, or enduring the p38MAPK cell signaling in mutant (mut) p53 gain-of-function scenario.

Accordingly, contradictive effects have been reported upon p38MAPK pathway modulation in cancer. In accordance with its p53 activating role, it has been proposed that p38MAPK activation could act as an oncosuppressive pathway; however, evidence also suggests that p38MAPK signaling is highly active in different types of cancers, favoring tumor growth [2,9,10,11].

While the different outcome of p38MAPK activation is relatively unsurprising, given its ability to mediate signaling in response to different and often antithetic stimuli, this also limits its therapeutical exploitation. In this perspective, modulating specific arms of the p38MAPK signaling pathway could represent a more effective strategy. Hence, a precise characterization of the different players involved in its signaling cascade could not only shed light onto the mechanisms underlying the different reported outcomes of p38MAPK activation, but also identify potential therapeutic targets. In this context, the mutp53 gain-of-function transcriptional target and p38MAPK upstream MAP2K, MKK3 has been recently proposed as a target for tumor therapy [12,13,14].

Here we summarize the current knowledge on MKK3/6-p38MAPK pathway and its delicate interaction with p53, revealing an intricate balance that could be shifted according to cell-type and —context.

## 2. TP53: Apoptosis and More

The TP53 tumor suppressive role has been classically attributed to its ability to work as a rheostat to induce cell-cycle arrest and promote DNA repair or initiate apoptosis in response to cellular stress. In fact, while physiologically low p53 levels are maintained via MDM2 mediated ubiquitination, upon DNA damage, p53-MDM2 binding gets disrupted causing p53 accumulation and activation, ultimately leading to p21-mediated cell-cycle arrest and/or senescence or Bax/PUMA/Noxa-mediated apoptosis. In cancer, the increased proliferative rate favors DNA damage, and inactivation of p53 becomes essential for cancer to arise and progress: in fact, *p53* mutation events are detected in approximately half of solid tumors. Mutations result into either loss-of-function of p53 or gain-of-functions. Consistently with a central role of p53 in tumor suppression, a plethora of studies confirmed that impaired (in the case of loss-of-function) or aberrant p53 activity (in the case of gain-of-functions) [15,16,17], leads to increased predisposition to tumor development and hence the restoring of physiological p53 activity is regarded as a possible therapeutical strategy [5,6,18].

Interestingly, while the tumor suppressive role of p53 remains a cornerstone, a series of studies using models defective for the activation of cell cycle inhibitors and proapoptotic mediators p21, PUMA and Noxa [19,20] demonstrated that the ability of p53 to act as a tumor suppressor does not rely on its ability to induce cell cycle arrest, senescence and/or apoptosis. Rather, p53 anti-tumor effect relies on its effects on metabolism, genetic and epigenetic stability, influence on inflammation, differentiation and integration with various signaling pathways [21].

In this new context, dysregulation of molecular pathways intersecting with p53, especially those with reported pleiotropic effects such as p38MAPK, could be reinterpreted to gain novel insights. Furthermore, delving into the exact contribution of each p53 mutation pattern could unveil new therapeutic avenues [6,22]. In particular, dissecting the role of *p53* mutations resulting into gain-of-function represents a peculiar challenge and with maybe an even greater reward, because, beyond affecting the different processes mediated by functional p53, they also modulate the activity of other pathways: understanding this cross-talk could theoretically offer highly selective interventional strategies.

## 3. MKK3/MKK6/P38 Signaling in Cancer: Friend or Foe

The p38MAPK pathway is used to respond and adapt to a wide variety of extracellular stimuli, including oxidative stress, UV irradiation, cytokines and growth factors [4,10].

To date, four p38MAPK isoforms, encoded by four different genes, have been identified: p38α and p38β, the two most studied isoforms, are very similar (75% homology) and are expressed in most tissues. p38γ and p38δ isoforms (sharing 70% homology among them and 60% with p38α, also referred to as alternative p38MAPKs [23]), have not been characterized as deeply as p38α and p38β, but have been brought to the spotlight more recently [23,24,25].

**Figure 1:**
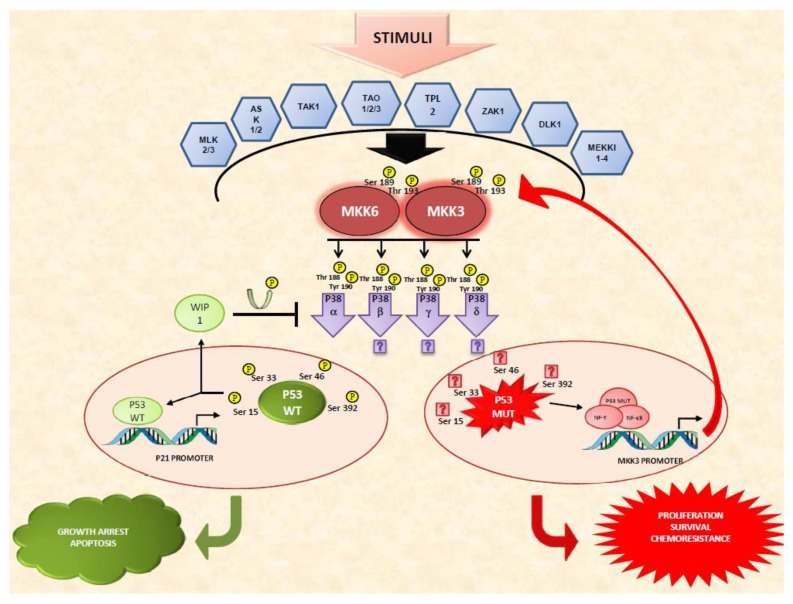
p53 and potential outcomes of p38MAPK activation. Both pro-apoptotic or survival stimuli can be transduced by p38MAPK pathway. A wide array of MAP3Ks stimuli that converge to MAP2Ks (MKK3 and MKK6) which, in turn, mediate the activation of different p38MAPK isoforms. Preferential activation of specific nodes and isoforms according to the type and/or duration of the stimulus are fundamental in the final biological outcome. In addition, cell-context and integration with other signaling pathways are able to skew the final outcome. In the wtp53 cell-context (**left**) activated p38MAPK alpha phosphorylates directly wtp53, contributing to its activation, and stabilize p21 mRNA orchestrating growth arrest or apoptosis. Conversely, activated wtp53 induces Wip1 phosphatase expression mediating a negative regulatory feedback on p38MAPK-p53 signaling. Conversely, in a mutant p53 cell-context (**right**), the increased *MKK3* gene expression by mutated protein, through NF-Y and NF-κB transcription factors, could contribute in sustaining and/or enhancing the p38MAPK signaling in a positive regulatory feedback, which potentially supporting further mutant p53 gain-of-function activities and thus cancer cell proliferation, survival or chemoresistance.

Beyond mechanisms involving autophosphorylation, which have been reported for α and β isoforms [26,27,28], p38 activation canonically follows a MAPK signaling module, being phosphorylated by MAPK kinases (MAP2Ks) which, in turn, are phosphorylated by MAPK kinase kinases (MAP3K). Specifically, for p38MAPK, different MAP3K, including ASK1/2, TAK1, TAO1/2/3, TPL2, MLK2/3, MEKK1-4, DLK-1 and ZAK1 converge to the same p38 specific MAP2Ks activators, i.e., MKK3 and MKK6 [1,9] (and in response to UV irradiation, the c-Jun N-terminal kinase (JNK) activator MKK4 [29]) (Figure 1). Although a considerable degree of functional overlap for the two main MAP2K exists, and compensatory mechanisms have been described [30], a differential role for either MKK3 or MKK6, according to the cellular context, has been reported [31,32]. Furthermore, experiments using knockout fibroblasts for either *MKK3* or *MKK6*, demonstrated a differential activation of the two MAP2Ks according to the type of stimulus as well as a specificity in the activation of different p38 isoforms. MKK3 and MKK6 are activators of p38α and p38β in response to environmental stress, MKK6 activates isoform p38γ in response to TNFα and MKK3 mediates p38δ activation as a consequence of ultraviolet radiation, hyperosmotic shock, anisomycin or TNFα exposure [33].

As for the two upstream MAP2Ks, also the role of the four p38 isoforms, although sharing a good size of both activating stimuli and downstream effectors, is distinct, with reported isoform-specific activation patterns, modulators, substrates and in some cases, antagonizing effects [23,25,34,35,36,37,38]. This intricate scenario of redundant functions on one hand, and different degrees of specificity in substrate activation on the other, is mirrored by the downstream modulation of a wide variety of targets, including protein kinases, phosphatases, transcription factors, and cell-cycle regulators [1,4]. Such a complex network has attracted many efforts at elucidating the therapeutic relevance of p38MAPK pathway modulation in several contexts. However, although p38MAPK inhibitors reached clinical testing (see www.clinicaltrials.gov and Table 1) also for cancer patients [39], their application presents serious challenges because of the pleiotropic nature of the p38MAPK pathway itself, with reports of p38MAPK exerting both pro-tumorigenic and oncosuppressive roles depending on cell-type and context [10,40,41,42] and affecting tumorigenesis by mediating inflammation [43]. Indeed, while pursuing a more tailored exploitation of p38MAPK inhibitors by identifying particular cell contexts in which inhibition of the whole pathway [44] or of specific isoforms [25,37,40] is therapeutically desirable, selective targeting of upstream signals represents an attractive alternative. In fact, blocking upstream kinases could interfere with specific p38MAPK isoforms and/or signaling modules, resulting into specific blocking of pro-tumorigenic signals and leaving tumor suppressive signals unaffected. In this perspective, isoform specific p38 inhibitors have been screened [45]. Similarly, also MKK6 and MKK3 MAP2Ks are currently being studied for the design of inhibitors that could potentially offer a targeted inhibition [46].

Unsurprisingly, even for these upstream targets, results seem to vary in different cell-contexts. In fact, MKK3 role in cancer appears controversial with reports of it favoring tumor growth [12,13,47] and mediating chemioresistance in lung cancer [32], but also acting as a tumor suppressor with reduced expression in malignant cells [48]. Similarly, also MKK6 appears to have a dual role: *MKK6* is overexpressed in esophageal, stomach and colon cancer [49] and increased expression has been observed in prostate cancer upon progression [50]. Additionally, MKK6 has been shown to mediate p38MAPK activation as a consequence of *H-Ras* expression in breast cancer cell lines [51]. However, MKK6 is also reported to act as a tumor suppressor, inhibiting cancer growth and metastasis [52,53].

These observations point towards the use of selective intervention on p38MAPK signaling pathway members only in specific cell contexts. In addition they also underscore the concept that effective exploitation of new inhibitors must be accompanied and guided by a deeper characterization of the pathway, its response to different stimuli and its interaction with other signaling routes.

## 4. Two Pathways Intersect: What Is the Outcome

The amount of contradictive data obtained about an unequivocal role of p38MAPK signaling in cancer, highlights how cell specific phenomena are able to skew the biological outcome of the activation of this specific pathway. Hence, a likely key for a targeted intervention on p38MAPK pathway, could lie in the dissection and characterization of its interaction with other signaling pathways, which could not only contribute to and/or modulate p38MAPK activity itself, but could also provide additional survival, proliferation, or death signals, driving the final outcome of p38MAPK activation.

In this regard, while the integration among different MAPK and non-MAPK pathways has long been hypothesized [2,4,54,55], characterization of this link remains elusive. In fact, despite the identification of always novel interactions among nodes of different pathways (e.g., Ras and MKK6 [34,51], MEK2 and p38MAPK [47], Myc and MKK3 [56]), including also non-MAPK pathways (e.g., MKK6/p38MAPK and IGF-1/Pi3K/Akt [57]), the extent to which the contribution of such links could be relevant in contexts other than the specific one in which they have been identified, still has to be fully elucidated. As an example, JNK and p38MAPK pathways appear to have mutually exclusive roles in some contexts [58] while they highly integrate in others [59,60]. It is also likely that only specific isoforms of p38MAPK and/or interacting proteins participate in specific nodes among pathways as reported for p38δ mediated senescence in the presence of oncogenic Ras [34,61].

Although the highly specific cell-contexts of these interactions perspectively restrict the use of targeted drugs to limited and well-defined cases, this huge and essential effort, is far from being unfruitful and provides clinicians with always new interventional strategies that go along well with the need for individualized therapies. Indeed, stratifying patients according to the cellular context that would allow for the best benefit from a given therapy is an ever more attractive goal. In the case of p53, the extremely high frequency of mutation would grant therapeutic intervention according to p53 status to benefit a large fraction of the patients.

In this perspective p38MAPK is able to control p53 activation, by direct phosphorylation [29,62,63], and p21 by stabilization at the mRNA level [64]. Conversely, p53-inducible wip1 phosphatase mediates a negative feedback regulation of p38MAPK-p53 signaling in response to UV radiation [62]. Consistently, the action of AGR2 oncoprotein was linked to inhibition of p53 phosphorylation by p38MAPK [65] and decreased p38α levels were linked to 5-FU innate and de novo resistance in colon cancer [66]. Similarly, ATAD-2 mediates MKK3/6 inhibition preventing p38MAPK activation, and ATAD-2 targeting resulted into p53 mediated apoptosis in hepatocellular carcinoma [67]. On the other hand, it is also reported that increased MKK3 levels are induced by mutant p53 transcriptional gain-of-function activity and contribute to growth in breast and colon tumor models [12,13]; moreover, targeting MKK3 triggers endop4lasmic reticulum stress and autophagic cell death promoting mutant p53 degradation and wild-type p53 stabilization [12]. In addition, p38MAPK/AP kinase-2 (MK-2) was demonstrated to be responsible for cisplatin resistance in p53 deficient cells, indicating simultaneous targeting of p53 and MK-2 could be exploited to induce synthetic lethality in lung cancer [68,69].

Again, the observed dual role for p38MAPK pathway activation could be ascribed to different cell contexts, de-regulated compensatory pathways, and the existence of regulatory feedbacks, as described for Wip1 [70,71], suggesting that, although not sufficient to depict the whole scenario, inserting other details (functional/defective or mutated p53) in the p38MAPK signaling pathway picture is at least enough to better define its boundaries (Figure 1).

In addition, both p38MAPK and p53 have been implicated in the regulation of inflammatory and immune responses [72,73], although further studies are needed to better define the relevance of their interaction in this perspective. In this regard, small molecules restoring p53 activity, such as PRIMA-1 and PRIMA-1^Met^, have shown promising results both alone and in combination with other treatments [74]. Investigating their potential also in the context of p38MAPK pleiotropic effects could reveal new fascinating scenarios.

## 5. Concluding Remarks

The tumor suppressor TP53 is a powerful transcription factor that upon activation protects organisms from propagation of cells carrying damaged DNA with potentially oncogenic mutations. In response to genotoxic or nongenotoxic stress, p53 is stabilized and activated by a complex network of post-translational modifications required to control cell cycle arrest, DNA repair, apoptosis, senescence, and autophagy. *TP53* gene is highly mutated in human cancer, differently from other tumor suppressors, mostly by missense mutation with accumulation of mutated protein that acquires novel or altered functions. Of interest, most p53 mutants can be post-translationally modified at the same residues as the wild-type protein. These modifications might alter mutant p53 activity contributing to gain-of-function. Hence, the understanding of signaling pathways that result in p53 modification and their impact of protein functions in both wild-type and mutant forms may contribute to more effective cancer therapies.

The p38MAPK is a major orchestrator of physiological and pathological cellular signaling and hence, an extremely attractive target for therapies. Indeed, correct targeting of this pleiotropic node, although desirable, needs to be accurately tailored in order to both trigger the anticipated response and avoid serious side-effects. Actually, considering the plethora of often contradictory functions that it exerts, obtaining a specific effect by modulation of the p38MAPK pathway is more like hitting a moving target. As a consequence, clinical testing of p38MAPK inhibitors, despite being applied into very specific contexts, could not meet expected results. In fact, research efforts have been focused to further define the cell-specific contexts in which modulation of the p38MAPK pathway could result into safe and effective therapeutic intervention and the development of selective inhibitors. However, this journey can not be disconnected from, and instead needs to be assisted by, a deeper characterization of the molecular players (isoforms, interacting pathways, modulators, activators) responsible for the biological outcome resulting from p38 activation.

In this regard, the big challenge of understanding the interaction of different members and isoforms of the p38MAPK signaling pathway and their integration with other signals from MAPK pathways, oncogenic and tumor suppressive mediators (such as p53), comes with the potential reward of a broad repertoire of therapeutic targets.

## Figures and Tables

**Table 1:** Clinical trials exploiting p38 inhibitors in cancer.

Study	Agent	Secondary Agent(s)	Target	Disease	Phase	Status
NCT01463631	LY3007113	N/A	p38	Metastatic cancer	I	Completed
NCT02364206	LY2228820	TMZ, Radiotherapy	p38	Glioblastoma	II	Active
NCT01663857	LY2228820	Carboplatin, Gemcitabine	p38	Ovarian cancer	II	Active
NCT02322853	LY2228820	Tamoxifen	p38	Breast cancer	II	Terminated
NCT01393990	LY2228820	N/A	p38	Advanced cancer	I	Completed
NCT02860780	LY2228820	Prexasertib	p38	Colorectal cancer, NSCLC	I	Completed
NCT00095680	SCIO-469	Bortezomib	p38	Multiple Myeloma	II	Completed
NCT00087867	SCIO-469	Bortezomib	p38	Multiple Myeloma	II	Completed
NCT00113893	SCIO-469	N/A	p38	Myelodysplastic syndrome	II	Completed
NCT01496495	ARRY-614	N/A	p38/Tie2	Myelodysplastic syndrome	I	Completed
NCT00916227	ARRY-614	N/A	p38/Tie2	Myelodysplastic syndrome	I	Completed
